# A Fractional Diffusion Model for Dye-Sensitized Solar Cells

**DOI:** 10.3390/molecules25132966

**Published:** 2020-06-28

**Authors:** B. Maldon, N. Thamwattana

**Affiliations:** School of Mathematical and Physical Sciences, University of Newcastle, Callaghan, NSW 2308, Australia; natalie.thamwattana@newcastle.edu.au

**Keywords:** dye-sensitized solar cells, electron density, fractional diffusion, subdiffusion, titanium dioxide, mathematical modelling

## Abstract

Dye-sensitized solar cells have continued to receive much attention since their introduction by O’Regan and Grätzel in 1991. Modelling charge transfer during the sensitization process is one of several active research areas for the development of dye-sensitized solar cells in order to control and improve their performance and efficiency. Mathematical models for transport of electron density inside nanoporous semiconductors based on diffusion equations have been shown to give good agreement with results observed experimentally. However, the process of charge transfer in dye-sensitized solar cells is complicated and many issues are in need of further investigation, such as the effect of the porous structure of the semiconductor and the recombination of electrons at the interfaces between the semiconductor and electrolyte couple. This paper proposes a new model for electron transport inside the conduction band of a dye-sensitized solar cell comprising of ${\mathrm{TiO}}_{2}$ as its nanoporous semiconductor. This model is based on fractional diffusion equations, taking into consideration the random walk network of ${\mathrm{TiO}}_{2}$. Finally, the paper presents numerical solutions of the fractional diffusion model to demonstrate the effect of the fractal geometry of ${\mathrm{TiO}}_{2}$ on the fundamental performance parameters of dye-sensitized solar cells, such as the short-circuit current density, open-circuit voltage and efficiency.

## 1. Introduction

Dye-sensitized solar cells (DSSCs) were first introduced by O’Regan and Grätzel in their fundamental 1991 paper [1], providing a viable low-cost alternative for renewable solar energy. Functionally, DSSCs operate by a photosensitive dye using absorbed sunlight to inject excited electrons into a nanoporous semiconductor. This approach relives DSSCs of the need for a costly and high purity semiconductor as opposed to the predominant silicon solar cells that have been at the forefront of solar energy since 1954 [2].

Mathematical modelling for DSSCs began shortly afterwards and continues to offer unique insight into the current–voltage relationship and ultimately the efficiency of the DSSC. While some early mathematical models of DSSCs used Maxwell’s equations and electric potentials [3], Gregg [4] outlined the prevalence of the photoinduced potential as more influential than electric fields in DSSCs. Södergren et al. [5] were the first to model DSSCs by a simple diffusion equation together with analytical expressions for the short-circuit current density $J_{sc}$ and the open-circuit voltage $V_{oc}$. Cao et al. [6] extended the model to include time-dependence, leading to the partial differential equation

(1) $$\frac{\partial n}{\partial t}=D_{0}\frac{\partial^{2}n}{\partial x^{2}}+\varphi \alpha e^{-\alpha x}-k_{R}\left(n\left(x,t\right)-n_{eq}\right),$$

where n(x,t) is the conduction band electron density at depth x∈[0,d] and time t≥0, $D_{0}$ is the diffusion coefficient, φ is the incident photon flux, α is the absorption coefficient, $k_{R}$ is the recombination constant and $n_{eq}$ is the equilibrium electron density.

Anta et al. [7] considered a fully nonlinear diffusion equation for DSSCs and investigated the effect of different power-law diffusivities, which was analysed using Lie symmetry by Maldon et al. 2020 [8]. Andrade et al. 2011 [9] and Gacemi et al. 2013 [10] proposed diffusion equations for modelling the electrolyte concentrations, which were solved analytically by Maldon and Thamwattana in 2019 [11].

Fractional calculus has existed conceptually as long as calculus itself. For close to three centuries, fractional calculus had only one known application in Abel’s 1823 tautochrone problem [12] until Nigmatullin [13] suggested a fractional diffusion equation for modelling media exhibiting a fractal geometry. In 2000, Henry and Wearne [14] developed the standard fractional diffusion equation based on continuous-time random-walk (CTRW) models. Henry and Wearne’s model [14], together with the CTRW simulation of ${\mathrm{TiO}}_{2}$ by Nelson [15], provides motivation for this paper to model DSSCs using fractional partial differential equations.

Modelling DSSCs with fractional calculus is a relatively unexplored research direction. So far, there is only one paper by Sibatov et al. in 2014 [16]. In Sibatov et al. [16], they consider the role of trap states within the ${\mathrm{TiO}}_{2}$ network and its effect on the electron hole density. To address the fact that previous mathematical models for DSSCs do not generally consider the effect of the porous network in ${\mathrm{TiO}}_{2}$ [6,17], we develop our model based on generalised fractional diffusion-reaction equations, taking this effect into account.

For this study, we use the Caputo fractional derivative, which is given by [18]

(2) $$\frac{d^{\lambda }f}{dt^{\lambda }}=\left\{\begin{array}{c} f^{\left(\lambda \right)}\left(t\right),\;\mathrm{if}\;\lambda \in N,\\ \int_{0}^{t}\frac{{\left(t-\tau \right)}^{\left(n-\lambda -1\right)}}{\Gamma (n-\lambda )}f^{\left(n\right)}\left(\tau \right)d\tau , \end{array}\right.$$

where λ>0 is the order of the fractional derivative, n=⌈λ⌉, $f^{\left(n\right)}\left(t\right)$ denotes the classical derivative of *f* with respect to its variable of order n∈N and Γ is the usual Gamma function

$$\Gamma \left(z\right)=\int_{0}^{\infty }x^{z-1}e^{-x}dx.$$

The literature employs several definitions for the fractional derivative in modelling diffusion in media exhibiting a fractal geometry. Henry and Wearne [14] suggest the Riemann–Liouville definition, but also remark that the Caputo definition has also seen some use [14,19]. However, Baeumer et al. [20] comment on the use of Caputo derivatives for space-fractional diffusion models that positivity is not preserved under vanishing Neumann boundary conditions. We find that problem is alleviated by the use of a time-fractional derivative on the diffusion term of our equation. Also, the presence of spatially dependent source terms and the combination of Dirichlet and Neumann boundary conditions enjoy a greater level of compatibility with the Caputo fractional derivative [21].

## 2. Mathematical Model

In this paper, we adopt a Caputo fractional derivative in time on the diffusion term as shown by Henry and Wearne [14], resulting in the fractional partial differential equation (FPDE)

(3) $$\frac{\partial n}{\partial t}=D_{0}\frac{\partial^{1-\gamma }}{\partial t^{1-\gamma }}\left(\frac{\partial^{2}n}{\partial x^{2}}\right)+\varphi \alpha e^{-\alpha x}-k_{R}\left(n\left(x,t\right)-n_{eq}\right),$$

where γ∈(0,1] is the order of the Caputo fractional derivative in time and all other parameters retain their values as in Equation (1). Physically, the parameter γ is the exponent in the mean square displacement of the CTRW simulation [14]. The special case γ=1 recovers the standard diffusion equation. Lower values for γ correspond to longer path lengths for electron transport through the nanoporous semiconductor [22]. The parameter γ is also strongly influenced by the porosity of the ${\mathrm{TiO}}_{2}$ semiconductor. Benkstein et al. [22] found that increasing porosity led to a decrease in γ. We note that Equation (3) does not feature the term

$$D_{0}L^{-1}\left(\frac{\partial^{-\gamma }}{\partial t^{-\gamma }}\frac{\partial^{2}n}{\partial x^{2}}{|}_{t=0}\right),$$

where $L^{-1}$ denotes the inverse Laplace transformation. Though this term is critical for physically meaningful fractional diffusion equations [14], it vanishes in Equation (3) under the Caputo fractional derivative.

We prescribe boundary conditions as found in [5], with a Dirichlet boundary condition at x=0 and a Neumann boundary condition at x=d together with a prescribed initial condition, namely

(4) $$\begin{array}{c} n\left(0,t\right)=n_{eq}e^{\frac{qV}{mk_{B}T}}, \end{array}$$

(5) $$\begin{array}{c} \frac{\partial n}{\partial x}{|}_{x=d}=0, \end{array}$$

(6) $$\begin{array}{c} n\left(x,0\right)=n_{eq}e^{\frac{qV}{mk_{B}T}}, \end{array}$$

where *q* is the standard electron charge, *V* is the bias voltage, *m* is the diode ideality factor, $k_{B}$ is Boltzmann’s constant and *T* is the temperature of the DSSC.

The diode equation is commonly used to compute the current–density relationship for solar cells, in which the current *J* as a function of bias voltage *V* is given by

(7) $$J\left(V\right)=J_{sc}-J_{0}\left(e^{\frac{qV}{mk_{B}T}}-1\right),$$

where $J_{0}$ is the dark saturation current density, given by Södergren et al. [5] in the form

$$J_{0}=qn_{eq}\sqrt{D_{0}k_{R}}\tanh\left(\sqrt{\frac{k_{R}}{D_{0}}}d\right).$$

To compute the short-circuit current density $J_{sc}$ we use

(8) $$J_{sc}=qD_{0}\left[\frac{\partial^{1-\gamma }}{\partial t^{1-\gamma }}\left(\frac{\partial n}{\partial x}\right)\right]{|}_{x=0},$$

noting that the standard flux is recovered in the special case of linear diffusion (γ=1).

Given that the open-circuit voltage $V_{oc}$ satisfies $J(V_{oc})=0$, we may compute the open-circuit voltage from

$$V_{oc}=\frac{mk_{B}T}{q}\ln\left(\frac{J_{sc}}{J_{0}}+1\right).$$

Maximising the power output P(V)=VJ(V) over *V*, we obtain the maximum power point $V_{max}$ by

$$V_{max}=\frac{mk_{B}T}{q}\left(W\left(e\frac{J_{sc}+J_{0}}{J_{0}}\right)-1\right),$$

where *W* is the Lambert-W function and $J_{max}=J\left(V_{max}\right)$. With $P_{max}=V_{max}J_{max}$, we compute the efficiency η of the DSSC by

$$\eta =\frac{P_{max}}{P_{i}},$$

where $P_{i}$ is the power of incident light.

## 3. Finite Difference Method

Finite difference methods (FDMs) have been used by Hu et al. [23] to solve parabolic FPDEs under the Caputo fractional derivative for fractional time derivatives, and Takeuchi et al. [24] have used finite difference methods to solve FPDEs under fractional spatial derivatives. We refer the reader to Li and Zeng [25] for a finite difference scheme for solving fractional ordinary differential equations over a finite interval under boundary conditions defined at the left boundary.

In this paper, we solve Equation (3) under a FDM scheme using expressions given by Oldham and Spanier [26]. All numerical computations are performed using numerical values of constants provided in Table 1 except for γ, which is given several values in Table 2.

To numerically solve Equation (3) under boundary conditions (4)–(6) with a finite difference scheme, we use the L1 approximation for the fractional derivative given by Oldham and Spanier [26] in which

(9) $$\frac{\partial^{\gamma }f}{\partial t^{\gamma }}{|}_{t=t_{n}}\approx \frac{{\left(\Delta t\right)}^{-\gamma }}{\Gamma (2-\gamma )}\sum_{k=0}^{n-1}b_{k}^{\gamma }\left[f\left(t_{n-k}\right)-f\left(t_{n-k-1}\right)\right],$$

where $b_{k}^{\gamma }$ is given by

$$b_{k}^{\gamma }={\left(k+1\right)}^{1-\gamma }-k^{1-\gamma }.$$

Let $t_{f}>0$ be the final simulation time. Discretise $\left[0,t_{f}\right]$ into $N_{t}$ nodes and [0,d] into $N_{x}+1$ nodes, and let $u_{i,j}$ estimate the solution to Equation (3) under the boundary conditions (4)–(6) at the point ((j−1)Δx,(i−1)Δt). That is,

(10) $$n\left(\left(j-1\right)\Delta x,\left(i-1\right)\Delta t\right)\approx u_{i,j}.$$

### 3.1. Nodes Determined by Boundary Conditions

To satisfy the initial condition (6), we set

$$u_{1,j}=n_{eq}e^{\frac{qV}{mk_{B}T}},$$

for all $j\in \{1,\ldots ,N_{x}+1\}$. For the boundary condition (4) at x=0, we set

$$u_{i,1}=n_{eq}e^{\frac{qV}{mk_{B}T}},$$

for all $i\in \{1,\ldots ,N_{t}\}$. Finally, for the Neumann boundary condition (5) at x=d we employ a ‘ghost node’ at x=d+Δx and a central difference approximation for the first derivative at x=d to set

$$u_{i,Nx+1}=u_{i,Nx-1},$$

for all $i\in \{1,\ldots ,N_{t}\}$.

### 3.2. Iteration Algorithm

For the second row i=2, we have

$$\begin{array}{cc} u_{2,j} & =u_{1,j}+\left(\Delta t\right)\varphi \alpha e^{-\alpha (j-1)(\Delta x)}-k_{R}\left(u_{1,j}-n_{eq}\right)\\ \; & =n_{eq}+\left(\Delta t\right)\varphi \alpha e^{-\alpha (j-1)(\Delta x)}. \end{array}$$

Given $i\in \{2,\ldots ,N_{t}\}$ and j∈{2,…,Nx}, the finite difference iteration for numerically solving Equation (3) is given by

(11) $$\begin{array}{c} u_{i+1,j}=u_{i,j}+D_{0}\frac{{\left(\Delta t\right)}^{\gamma }}{\Gamma (\gamma +1)}\sum_{k=0}^{i-2}\left[{\left(k+1\right)}^{\gamma }-k^{\gamma }\right]\times \\ \left(\frac{u_{i-k,j+1}-2u_{i-k,j}+u_{i-k,j-1}-u_{i-k-1,j+1}+2u_{i-k-1,j}-u_{i-k-1,j-1}}{{\left(\Delta x\right)}^{2}}\right)\\ +\left(\Delta t\right)\left[\varphi \alpha e^{-\alpha (j-1)(\Delta x)}-k_{R}\left(u_{i,j}-n_{eq}\right)\right]. \end{array}$$

### 3.3. Estimate for Short-Circuit Current Density

To compute the short-circuit current density, we must estimate the electron flux at x=0. We achieve this by a 10 point estimate of the form

(12) $$\begin{array}{c} \frac{\partial n}{\partial x}{|}_{x=0}\approx a_{0}n\left(0\right)+a_{1}n\left(\left(\Delta x\right)\right)+a_{2}n\left(2\left(\Delta x\right)\right)+a_{3}n\left(3\left(\Delta x\right)\right)+a_{4}n\left(4\left(\Delta x\right)\right)\\ +a_{5}n\left(5\left(\Delta x\right)\right)+a_{6}n\left(6\left(\Delta x\right)\right)+a_{7}n\left(7\left(\Delta x\right)\right)+a_{8}n\left(8\left(\Delta x\right)\right)+a_{9}n\left(9\left(\Delta x\right)\right), \end{array}$$

where $a_{i}$ is a constant for each i=0,1,…,9. We determine these constants so that polynomials are perfectly estimated up to degree 9. This leads to the constants

$$\begin{array}{c} a_{0}=-\frac{7129}{2520(\Delta x)},a_{1}=\frac{9}{\left(\Delta x\right)},a_{2}=-\frac{18}{\left(\Delta x\right)},a_{3}=\frac{28}{\left(\Delta x\right)},a_{4}=-\frac{63}{2(\Delta x)},\\ a_{5}=\frac{126}{5(\Delta x)},a_{6}=-\frac{14}{\left(\Delta x\right)},a_{7}=\frac{36}{7(\Delta x)},a_{8}=-\frac{9}{8(\Delta x)},a_{9}=\frac{1}{9(\Delta x)}. \end{array}$$

## 4. Results and Discussion

**Figure 1:**
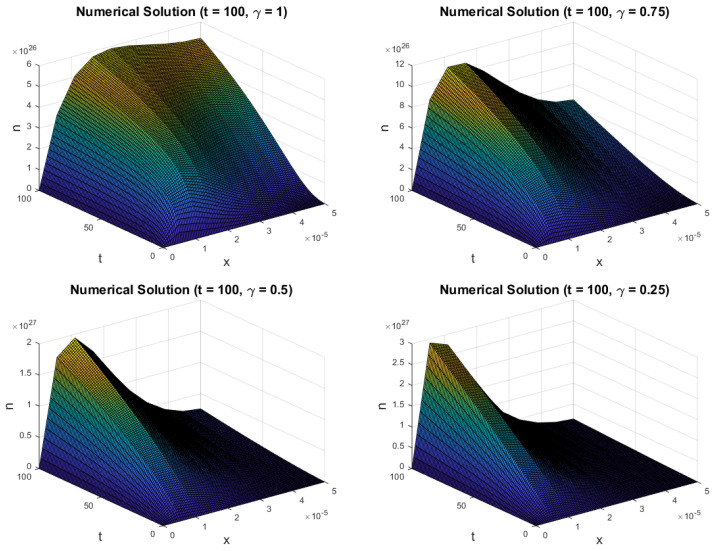
Plots of the numerical solution to Equation (3) against *x* and *t* for γ=1 (top-left), γ=0.75 (top-right), γ=0.5 (bottom-left), γ=0.25 (bottom-right).

Using the finite difference method, we numerically solve Equation (3) for several values of γ using 10 spatial nodes and 5000 temporal nodes over x∈[0,d] and t=100 s. To investigate the effect of the parameter γ, we plot numerical solutions for the special cases γ=0.25,0.5,0.75and1 in Figure 1.

Using the numerical solutions to Equation (3) and the flux estimate given by Equation (12), we are able to compute the short-circuit current density $J_{sc}$ and the open-circuit voltage $V_{oc}$, leading to the overall efficiency η. Table 2 shows the effect of the parameter γ on these DSSC performance parameters.

From Table 2, we see that the short-circuit current density $J_{sc}$ increases when γ increases. Though the open-circuit voltage is not affected to the same extent, the efficiency is notably lower for decreased values of γ. We note the special case γ=1 is equivalent to the standard diffusion equation without fractional derivatives, and the efficiency η=7.03% is in agreement with expected efficiencies for DSSCs [8].

As γ decreases, Figure 1 presents two primary trends to the numerical solution to Equation (3). Firstly, the time required to reach steady-state increases as γ decreases by comparison to the numerical solutions for the cases γ=1and0.25. This result is consistent with the observation that lower values for γ imply slower diffusion, based on the CTRW simulations. Benkstein et al. [22] show that γ decreases when porosity increases from p=0.7 to p=0.775.

Secondly, the overall electron density is remarkably higher for the cases γ=0.5and0.25 compared to γ=1and0.75. This suggests that the electron density is highly sensitive to the order of the fractional derivative. The standard gradient for flux would consequently produce extreme results, requiring the fractional derivative to rectify this issue. Numerically, the finite difference scheme presents stability problems and is computationally expensive for lower values of γ.

The parameter γ denotes the exponent for the power-law in the mean-square displacement [14]. We conclude from Figure 1 that lower values of γ lead to progressively less realistic behaviour for nanoporous semiconductors used in DSSCs, as the electron density dramatically increases when γ decreases. This observation is consistent with the longer path lengths associated with low values for γ in CTRW simulation. In particular, Ni et al. [27] show that extremely low porosities (such as p=0.1) show a significant reduction in efficiency (below 1%). From the numerical solution to Equation (3) with γ=0.612 using 25 spatial nodes, the flux estimate given by Equation (12) and 50,000 temporal nodes to t=500 s, we find $J_{sc}=116.9059Am^{-2}$, $V_{oc}=0.6245V$ and η=6.0835%.

## 5. Conclusions

We propose a new mathematical model for evaluating the efficiency of dye-sensitized solar cells by using fractional diffusion to incorporate the fractal geometry of the ${\mathrm{TiO}}_{2}$ semiconductor. Our results show that lower values of the mean square exponent γ lead to lower efficiencies, a result that is consistent with the literature [11,27]. In particular, Figure 11 of Ni et al. [27] shows that efficiency decreases when porosity increases above p=0.4 or decreases below p=0.4, which suggests the relationship between porosity and efficiency is nonlinear. We note that the solution profile of the electron density presented here is similar to those obtained from nonlinear diffusion modelling [8], though the orders of magnitude differ significantly. This is due to the longer waiting times associated with lower values for γ, which slows down the diffusion process.

We also develop a finite difference scheme to numerically solve the fractional diffusion equation and provided a tenth-order estimate for obtaining the short-circuit current density. Together, this provides a comprehensive model for incorporating the effect of the random-walk behaviour of the nanoporous semiconductor on the performance of dye-sensitized solar cells.

Future consideration includes incorporating the role of the electrolyte couple by a pair of standard diffusion equations as seen in Maldon and Thamwattana [11].

## Figures and Tables

**Table 1:** Parameter values for the Dye-Sensitized Solar Cell (DSSC) model.

Parameter	Value	Unit	Reference
$D_{0}$	$10^{-11}$	$m^{2}s^{-1}$	[7]
α	$10^{5}$	$m^{-1}$	[10]
*d*	$5\times 10^{-5}$	m	[7]
$k_{R}$	$4\times 10^{-8}$	$s^{-1}$	[7]
*m*	1	-	[5]
$n_{eq}$	$10^{22}$	$m^{-3}$	[27]
$P_{i}$	10	$Wm^{-2}$	[10]
φ	$10^{21}$	$m^{-2}s^{-1}$	[28]

**Table 2:** Values for $J_{sc}$, $V_{oc}$ and η for several values of γ at time t=100 s.

γ	$J_{\mathrm{sc}}\;\left(Am^{-2}\right)$	$V_{\mathrm{oc}}\left(V\right)$	η(%)
0.25	56.0248	0.6056	2.8144
0.39	70.7900	0.6117	3.5967
0.5	82.6370	0.6156	4.23
0.612	94.6085	0.6191	4.8741
0.75	108.9019	0.6227	5.6481
1	134.2799	0.6281	7.03

## Abbreviations

The following abbreviations are used in this manuscript:

DSSC	Dye-Sensitized Solar Cell
FPDE	Fractional Partial Differential Equation
${\mathrm{TiO}}_{2}$	Titanium Dioxide
